# Bioinspired Remineralization of Artificial Caries Lesions Using PDMAEMA/Carbomer/Calcium Phosphates Hybrid Microgels

**DOI:** 10.3390/gels8100681

**Published:** 2022-10-21

**Authors:** Alexander Bonchev, Marin Simeonov, Pavletta Shestakova, Radosveta Vasileva, Rositsa Titorenkova, Anton Apostolov, Elena Dyulgerova, Elena Vassileva

**Affiliations:** 1 Faculty of Dental Medicine, Medical University, 1, G. Sofiiski Str., 1431 Sofia, Bulgaria; 2 Laboratory on Structure and Properties of Polymers, Faculty of Chemistry and Pharmacy, University of Sofia, 1, James Bourchier Blvd., 1164 Sofia, Bulgaria; 3 Institute of Organic Chemistry with Centre of Phytochemistry, Bulgarian Academy of Sciences, Acad. G. Bonchev Str., Bl. 9, 1113 Sofia, Bulgaria; 4 Institute of Mineralogy and Crystallography, Bulgarian Academy of Sciences, Acad. G. Bonchev Str., Bl. 107, 1113 Sofia, Bulgaria

**Keywords:** remineralization, dental caries, hybrid microgels, PDMAEMA, poly(acrylic acid)

## Abstract

Dental caries remains one of the most prevalent bacterium-caused chronic diseases affecting both adults and children worldwide. The development of new materials for enhancing its remineralization is one of the most promising approaches in the field of advanced dental materials as well as one of the main challenges in non-invasive dentistry. The aim of the present study is to develop novel hybrid materials based on (PDMAEMA)/Carbomer 940 microgels with in situ deposited calcium phosphates (CaP) and to reveal their potential as a remineralization system for artificial caries lesions. To this purpose, novel PDMAEMA/Carbomer 940 microgels were obtained and their core–shell structure was revealed by transmission electron microscopy (TEM). They were successfully used as a matrix for in situ calcium phosphate deposition, thus giving rise to novel hybrid microgels. The calcium phosphate phases formed during the deposition process were studied by X-ray diffraction and infrared spectroscopy, however, due to their highly amorphous nature, the nuclear magnetic resonance (NMR) was the method that was able to provide reliable information about the formed inorganic phases. The novel hybrid microgels were used for remineralization of artificial caries lesions in order to prove their ability to initiate their remineralization. The remineralization process was followed by scanning electron microscopy (SEM), X-ray diffraction, infrared and Raman spectroscopies and all these methods confirmed the successful enamel rod remineralization upon the novel hybrid microgel application. Thus, the study confirmed that novel hybrid microgels, which could ensure a constant supply of calcium and phosphate ions, are a viable solution for early caries treatment.

## 1. Introduction

Biomineralized hard tissues, such as bones and teeth, possess outstanding mechanical properties related to their composition as well as to their architecture and hierarchically arranged internal nanostructure [1]. For example, mature enamel consists of a ~96 wt.% highly organized mineral part with a unique structure where hydroxyapatite elongated nanocrystals are arranged roughly parallel to each other along the enamel rod c-axis [2,3]. Being non-living acellular tissue, the enamel does not self-repair after substantial mineral loss [4], therefore, dental caries is one of the most prevalent chronic diseases worldwide [5]. Thus, recently, enamel has been a subject of active research aiming at its regeneration via controlled remineralization [4].

Enamel caries pathophysiology is not simply a cumulative loss of enamel minerals, but rather a dynamic process characterized by discontinuous periods of demineralization and remineralization. While the enamel demineralization is a process of crystal dissolution, the remineralization is the restoration of the partially dissolved hydroxyapatite crystals via epitaxial growth of new crystal [6]. The delicate balance between de- and remineralization is controlled, on the one hand, by pathological factors such as cariogenic bacteria, fermentable carbohydrates and salivary dysfunction and, on the other hand, by protective factors such as saliva, antibacterial agents, remineralizing ions, etc. The balance between both processes determines the lesion progression as well as the reversal of the carious process [7]. In this respect, it is now well established that caries, in the early stage of its formation (i.e., when non-cavitated), can be repaired via remineralization [8] which shifts the focus of the biomineralization-based therapies to regenerative approaches using the biomimetic remineralization approach [9].

Remineralization is defined as the process where calcium and phosphate ions are supplied in the dental microenvironment from an external source to promote ion deposition into the crystal defects of the demineralized enamel, i.e., in defects with mineral loss. A better understanding of regenerative mechanisms of the remineralization process is expected to provide a relevant scientific pathway to develop a new innovative remineralization technology, going beyond the traditional fluoride-mediated dental remineralization [10,11,12]. For example, the fluoride-based approach results in a surface-zone remineralization [13] while the biomimetic strategies aim at a lesion full-body restoration. Most of the recent biomineralization therapies follow the amelogenin mode of action [14]. Extracellular matrix proteins, among which amelogenin is the most abundant one during the enamel secretory stage, act as nucleation templates in the biomineralization of enamel [15]. Due to the difficulties associated with extracting, purifying and storing this natural protein, there is an attempt to create a suitable synthetic analogue of amelogenin. Moreover, as an alternative strategy for bioinspired morphogenesis, the use of organic–inorganic materials and the application of polymer additives to modify the mineral crystallization is currently exploited. Thus, in the broadest sense, the bioinspired mineralization can be understood as the application of soluble polymeric additives (mimicking the protein matrix in natural enamel formation) for the control of crystallization reactions [16]. This approach is expected to be even more effective when the remineralization systems are combined with an antibacterial activity. To date, hybrid calcium phosphate materials based on linear polymers/biopolymers/peptides, etc., have been used to ensure higher calcium and phosphate ion concentrations in the vicinity of the caries, which are expected to enhance the caries remineralization. Following the idea of prolonged drug delivery, we aim to expand the concept of remineralization by creating a novel “reservoir” type delivery system for calcium and phosphate ions, based on polymeric microgels. Such a system is expected to ensure a continuous flux of calcium and phosphate ions released in the vicinity of the caries lesion which can enhance the successful enamel remineralization. Microgel structure is expected to play an important role in the performance which is why we explore in this study the core–shell type microgels and their calcium–phosphate hybrid materials as a new system for enamel remineralization enhancement.

Poly(N,N′-dimethylamino ethylmethacrylate) (PDMAEMA) is a cationic polymer which is water soluble and when dissolved in slightly acidic media (pK_b_~6.5) shows good antibacterial properties against Gram-positive and Gram-negative bacteria [17], including the main cariogenic pathogen in the human oral cavity, Streptococcus mutans [18]. Due to its charge, it is also characterized by good mucoadhesive properties [19]. Moreover, a greater content of PDMAEMA is reported to provide a better structural environment for the application of PDMAEMA-based materials as scaffolds for tissue engineering bone repair [20]. PDMAEMA is widely used as antibacterial agent and that is why, probably, PDMAEMA application in dental medicine has been limited to only its antibacterial activity to date, e.g., as a tool for fighting against the oral biofilm formation. We could not find literature data for PDMAEMA application, neither for caries prophylactics nor for enamel remineralization.

Carbomer 940, which is poly(acrylic acid) cross-linked with ethers of pentaerythritol, is widely used as a viscosity enhancer or gelling agent. It is an extremely efficient rheology modifier as it can provide high viscosity and can form clear gels and creams. Powdered Carbomers have a dry particle size of 2–7 microns, the particles being formed by the aggregation of smaller (50–300 nm) spherical microgel particles [21]. Although poly(acrylic acid) is used for enamel remineralization as linear homo- and copolymers, the Carbomer particles have never been reported for such an application. Moreover, we could not find in the literature any data about particles or microgels formed via Carbomer–PDMAEMA complexation, i.e., core–shell type structure.

The aim of the present study is to reveal the potential of novel hybrid PDMAEMA/Carbomer/calcium phosphate microgels as a remineralization system for artificial enamel lesions. To this purpose, PDMAEMA/Carbomer core–shell microgels were obtained and used as vehicles for in situ calcium phosphate deposition, thus giving rise to this novel hybrid material. The newly obtained hybrid materials were characterized in terms of their morphology (by transmission and scanning electron microscopy, TEM and SEM), CaP–polymer interactions (via infrared spectroscopy) as well as in terms of the CaP phases formed during their in situ formation (by NMR, X-ray and infrared spectroscopy). These materials were successfully applied for the remineralization of artificial caries lesions, thus demonstrating their potential for biomimetic remineralization of demineralized enamel.

## 2. Results and Discussion

### 2.1. Characterization of Hybrid PDMAEMA/Carbomer 940/CaP Microgels

#### 2.1.1. TEM Characterization of Hybrid PDMAEMA/Carbomer 940/CaP Microgels

**Figure 1:**
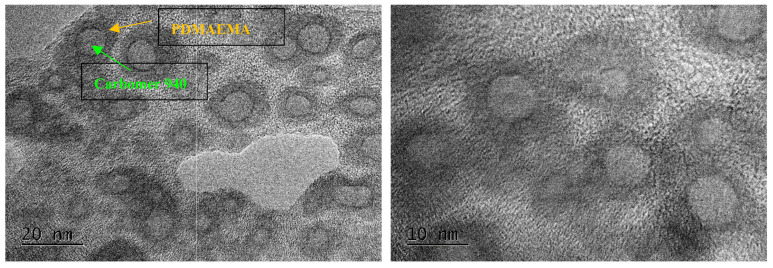
TEM image of hybrid PDMAEMA/Carbomer 940/CaP microgels.

The hybrid PDMAEMA/Carbomer 940/CaP microgels were studied by TEM (Figure 1). Carbomer 940 is known to be cross-linked poly(acrylic acid) with high molecular weight, forming spherical submicron particles. In Figure 1, these particles from Carbomer 940 are clearly seen as light circles, surrounded by a corona from PDMAEMA. The attachment of the positively charged PDMAEMA macromolecules to the negatively charged Carbomer 940 particles (pKa of the acrylic acid residues is ~5) is realized through electrostatic interactions, similarly to the mechanism of formation of polymeric layer-by-layer surfaces and particles. As far as we are aware, such core–shell microgels from PDMAEMA/Carbomer 940 are novel and have not been described to date.

In Figure 1, the PDMAEMA shell is darker as compared to the Carbomer 940 core due to the CaP deposition which takes place in the particles’ outer shell, i.e., in the corona, as it is more accessible as compared to the particle core—the inorganic part (CaP) has a higher density as compared to the polymers and that is why it looks darker in TEM.

The composites developed by the in situ precipitation of CaP within the core–shell PDMAEMA/Carbomer 940 microgels are also a novel material neither obtained nor explored to date, including for enamel remineralization.

#### 2.1.2. NMR

The chemical composition of the calcium phosphate phase formed in situ in the hybrid PDMAEMA/Carbomer 940/CaP microgels as well as without polymer was investigated by ^31^P solid state NMR spectroscopy. The CaP composition is expected to influence the remineralization ability of the hybrid microgels so its identification was important.

##### NMR Characterization of Neat CaP Phase (Obtained without Polymer)

**Figure 2:**
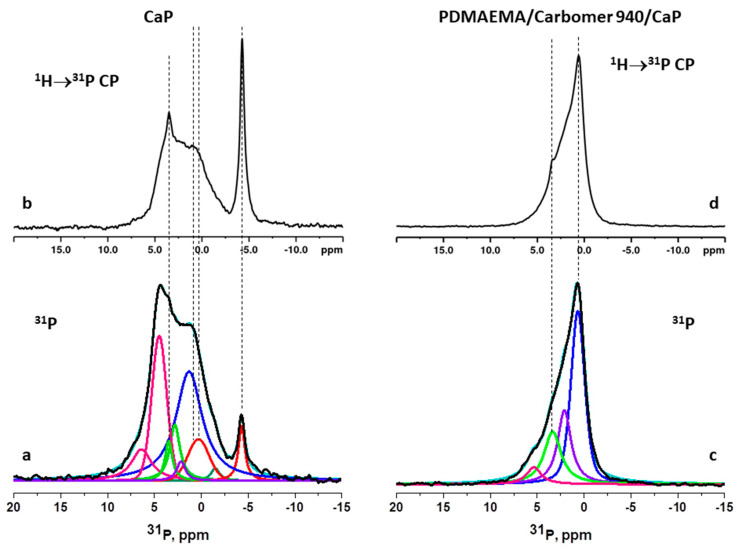
^31^P Single pulse and ^1^H→^31^P CP NMR spectra of CaP and PDMAEMA/Carbomer 940/CaP: (**a**) ^31^P NMR spectrum and (**b**) ^1^H→^31^P CP NMR spectrum of CaP; (**c**) ^31^P NMR spectrum and (**d**) ^1^H→^31^P CP NMR spectrum of PDMAEMA/Carbomer 940/CaP. The experimental single pulse ^31^P NMR spectra are presented with black lines and the simulated ones are given in light blue. The individual contributions of the different PO_4_^−^ environments are given with colored lines. The dotted lines indicate the resonances that are enhanced in the ^1^H→^31^P CP NMR spectra.

First, we studied the product of the reaction between CaCl_2_ and K_2_HPO_4_ under the experimental conditions used for the preparation of the hybrid polymer/CaP materials in order to obtain information about the formed calcium phosphate phase without polymer. This is presumed to help in better understanding the role of the polymers in the process of the CaP formation. The ^31^P NMR spectrum of the product obtained as a result of the model reaction is presented in Figure 2a.

##### Single Pulse ^31^P NMR Spectrum of the Hybrid PDMAEMA/Carbomer 940/CaP Microgels

The spectrum demonstrates a complex spectral pattern covering the region between −5 and 7 ppm, indicating the presence of both unprotonated orthophosphates (chemical shift region above 2 ppm) and hydrogen phosphate species (chemical shift region below 2 ppm). Using the characteristic chemical shift values from literature data, the spectral pattern was deconvoluted to the contributions of the different possible calcium phosphate phases. Table S1 summarizes the chemical shifts of the different calcium phosphates reported in the literature and used for the deconvolution of the spectral pattern of the mixed calcium phosphate phase obtained as a result of the reaction. The best fit between the experimental and simulated spectra was obtained when taking into account the spectral signatures of the following calcium phosphate phases: *monocalcium phosphate monohydrate* (MCPM, Ca(H_2_PO_4_)_2_.2H_2_O), *monetite* (CaHPO_4_), *dicalcium phosphate dihydrate* (Brushite, DCPD, CaHPO_4_.2H_2_O) and/or *amorphous calcium phosphate* (ACP, Ca_x_H_y_(PO_4_)_z_·nH_2_O), poorly crystalline *hydroxyapatite* (HAP, Ca_10_(PO_4_)_6_(OH)_2_*), amorphous tricalcium phosphate* (TCP, Ca_3_(PO_4_)_2_) and *octacalcium phosphate* (OCP, Ca_8_H_2_(PO_4_)_6_⋅5H_2_O).

The ^1^H→^31^P cross-polarization MAS (CP MAS) spectrum where the signals of ^31^P sites in close proximity to protons are selectively enhanced due to transfer of polarization from the neighboring protons provides further identification of the HPO_4_^2−^-containing phases (Figure 2b). The ^1^H→^31^P CPMAS experiment indicates that the most enhanced signals are those at −4.4 ppm and in the region at around 0 to 1 ppm for the acidic orthophosphates (such as MCPM and DCPA) as well as at 3.4 ppm for the ^31^P sites in close proximity to water molecules residing at the OH sites in the apatite structures.

##### NMR Characterization of CaP Phase Obtained via In Situ Precipitation in the Hybrid PDMAEMA/Carbomer 940/CaP Microgels

Figure 2c shows the single pulse ^31^P NMR spectrum of the hybrid PDMAEMA/Carbomer 940/CaP microgels. The deconvolution of the spectrum demonstrates the presence of at least four different structural environments of -PO_4_^3−^ groups with chemical shifts at 0.67 ppm, 2.10 ppm, 3.35 ppm and 5.35 ppm. Considering that the actual chemical shift values of the calcium phosphate species could be influenced by the interaction of the orthophosphate anions with the polymer matrix, we suggest that, most probably, in the microgels there is a mixture of amorphous TCP, HAP, ACP, OCP and/or DCPD. Thus, the presence of PDMAEMA/Carbomer 940 during the CaP formation seems to suppress the formation of acidic CaP as MCPM and monetite which are not detected in the obtained hybrid polymer/CaP microgels. This result is one of the first reported in the literature to date to estimate the influence of the polymer functionality on the type of CaP formed in situ. Most of the studies to date report the influence of pH over the CaP deposition—acidic pH of the medium where the CaP deposition takes place results in acidic CaP, while the alkaline pH usually results in apatite formation.

The broad resonances indicate formation of amorphous phases and heterogeneous structural environment of the orthophosphate species. Figure 2d presents the ^1^H→^31^P CPMAS spectrum of the hybrid PDMAEMA/Carbomer 940/CaP microgels. The spectral pattern is similar to the ^31^P single pulse spectrum and only a slight enhancement of the resonances at 0.5 ppm and at around 3.4 ppm was detected. This result indicates that the transfer of proton magnetization from the large pool of polymeric protons to the phosphorous species is very efficient even in short contact times and dominates over the magnetization transfer from the protons that are part of the orthophosphate structure. The overall analysis of the NMR spectra confirms the successful deposition of amorphous orthophosphate phase with both acidic as well as apatite CaP structures in the polymeric microgels.

#### 2.1.3. IR Spectra Characterization of PDMAEMA/Carbomer 940/CaP Hybrid Microgels

**Figure 3:**
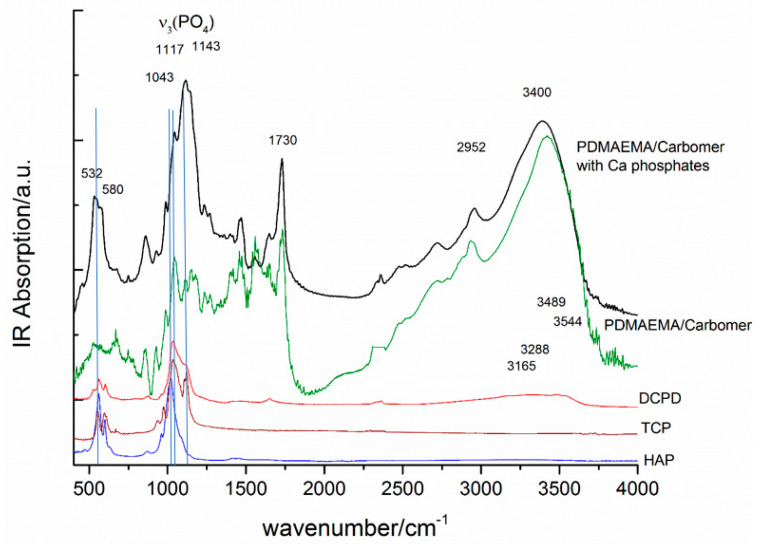
IR spectra of neat PDMAEMA/Carbomer 940 microgels as well as of the hybrid PDMAEMA/Carbomer 940/CaP microgels (IR spectra of DCPD, TCP and HAP are also provided for sake of comparison).

The infrared spectra of the neat PDMAEMA/Carbomer 940 microgels and their CaP hybrids are presented in Figure 3. The IR spectrum of PDMAEMA/Carbomer 940 microgels, collected in transmittance mode, reveals peaks which are characteristic for both polymeric components, namely poly(acrylic acid) (Carbomer 940) and PDMAEMA (see the IR characterization section in the Supplementary Information). These peaks coming from both polymers are also clearly seen in the spectrum of the hybrid microgels (Figure 3).

The formation of CaP phases in the hybrid materials is clearly evidenced by the strong intensity increase in the range 1000–1140 cm^−1^ which is characteristic for phosphate group stretching modes as well as in the range of 532–580 cm^−1^ where (PO_4_) bending vibrations are. Due to overlap of the peaks, typical for various calcium orthophosphates, the identification of particular phases is not unambiguous using only IR. That is why we provided in Figure 3 the infrared spectra of some of the phosphate phases identified by NMR and summarized the positions of the respective peaks for the calcium phosphate phases in Table S2 (Supplementary Information).

When comparing the data in Table S2 and Figure 3, it is seen that the absorption maximum in the spectrum of hybrid microgels is centered above 1100 cm^−1^ which is in the range characteristic for TCP, DCPD, OCP and ACP rather than in the range of apatite, whose highest absorption due to antisymmetric stretching is in the range of 1040–1090 cm^−1^. Thus, these phases probably predominate over apatite which is also supported by the positions of the bending vibration peaks at 532–580 cm^−1^—these are closer to those in HPO_4_ atomic groups, while in apatite such peaks are at higher frequencies at 560–603 cm^−1^ (Table S2, Supplementary Information). In the water stretching region, the peaks are overlapped by the most intensive absorption of polymer, which makes it difficult to identify water-containing phosphates.

Based on the IR spectra, it can be confirmed that a mixture of calcium orthophosphate phases is formed when CaP are in situ formed into PDMAEMA/Carbomer 940 microgels, as also determined by NMR.

The characterization of the hybrid PDMAEMA/Carbomer 940/CaP microgels by the methods presented above demonstrates that the calcium phosphate phases undergo transformations during the process of their formation depending on the conditions used. The kinetic pathway of CaP phase formation without polymer occurs via the classical nucleation theory (CNT) where the ions undergo stochastic collisions which lead to the formation of thermodynamically unstable pre-critical nuclei [22]. These nuclei can grow without limit when they reach a critical size, if a sufficient supply of ions is ensured. This critical size depends on the level of supersaturation. When more than one polymorph is accessible, as is the case with CaP, the fastest nucleation rate will determine which phase will be formed [23]. This, in fact, explains what is happening during the CaP formation, performed within the study, without polymer.

When the CaP deposition takes place in the presence of PDMAEMA/Carbomer 940 microgels, the formation of calcium phosphates occurs via the pre-nucleation cluster (PNC) pathway [7]. The ions form thermodynamically stable PNCs, which are rendered as phase-separated nanodroplets. These nanodroplets undergo aggregation and/or coalescence, yielding larger liquid intermediate phases which dehydrate and solidify, forming amorphous intermediates [7]. These intermediates could eventually transform into crystals if sufficient time for this process is ensured. In our case, the pre-nucleation clusters are solidified into amorphous metastable intermediates, which are further transformed into one of the most stable CaP phases, namely OCP, which is known to be the immediate precursor phase prior to HA formation. The transformations described above are all in line with the energetic pathways of biomineralization summarized recently [23]. These processes of nucleation and phase transformation, observed within the study, are controlled mostly by PDMAEMA as this is the polymer from the outer shell of the PDMAEMA/Carbomer 940 microgels (see Figure 1). All calcium phosphate phases identified by the NMR and IR methods in the newly obtained hybrid microgels are known to be precursors for apatite or hydroxyapatite formation. Thus, we hypothesize that the hybrid PDMAEMA/Carbomer 940/CaP microgels have a great potential as a biomimetic remineralization system.

### 2.2. Characterization of Demineralization and Remineralization of Enamel

The complex enamel architecture and the gradient in its composition play a unique role in the kinetics of enamel dissolution, i.e., demineralization. Numerous compositional, chemical and structural factors as well as the direction and the position of the acid attack also play role in lesion development [24]. Demineralization of samples was realized by using chemical models that mimic the caries process via the use of acid or acid buffer [25]. The main disadvantage of the chemical models is that they ignore the microbiological aspect of the caries formation [25]. Although the in vitro caries model does not entirely replicate the intraoral conditions, it is widely used and accepted to study the elementary steps in de- and remineralization processes. Artificial carious lesions were chosen to be used within this study as they are more reproducible when compared to the natural carious lesions. This is expected to make the caries models well set up and more reliable.

#### 2.2.1. SEM Characterization of Treated Tooth Surfaces

The surfaces of demineralized and remineralized artificial caries lesions were studied by SEM in order to reveal the hybrid microgel’s ability for enamel repair.

##### SEM Characterization of Artificial Caries Lesions

**Figure 4:**
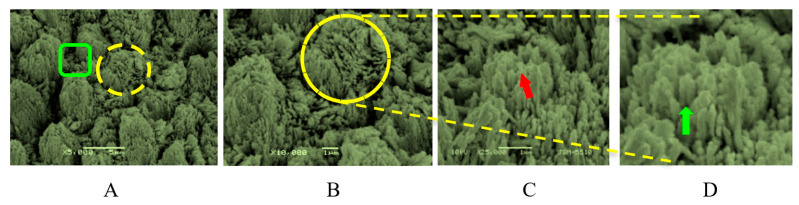
SEM study of demineralized tooth surface (artificial caries lesion): (**A**) HAP rods (yellow circle) and interrods (green rectangle) underwent inhomogeneous anisotropic dissolution along the *c*-axis; (**B**) HAP crystal arrangement in the rods; (**C**) and (**D**) HAP crystals are partially dissolved in their core (red arrow in (**C**)) and at their periphery (green arrow in (**D**)) (the images show one region of the artificial caries lesion at different magnifications).

The SEM images of demineralized tooth surface clearly reveal the fine enamel structure consisting in rods and interrods, both being built up by aligned carbonated HAP crystals (Figure 4). The carbonated HAP crystals have width of 50–70 nm, thickness of 20–25 nm and length to width aspect ratio over 1000 [26], i.e., their length is in the μm scale (Figure 4D, green arrow). They are aligned either into enamel rods with diameter ~5 μm (Figure 4A, yellow circle) or in interrods (Figure 4A, green rectangle).

The rods and interrods differ only in the orientation of the HAP crystals that build them up: the HAP crystals in rods are generally oriented parallel to the rod (Figure 4A, yellow circle), while the interrod HAP crystals are arranged at a 40 to 60° angle to the rod axis (Figure 4A, green rectangle). The border where the crystals of enamel rods and crystals of enamel interrods meet, also called a rod sheath, is where the organic part of the enamel is situated. It is not easily seen in Figure 4, as it is etched by the lactic acid treatment.

The dissolution of the enamel, as studied in carious as well as in acid-treated enamel, is a complex process, known to proceed preferentially in the rods’ core (along their long c-axis), as well as at their periphery [27,28,29]. The dissolution triggers the central perforation in HAP crystals, which means that the central part of HAP crystals is preferentially demineralized (Figure 4C) [28,29,30]. Upon increasing the magnification, the dissolution of the HAP crystals’ core along the crystal c-axis as well as the widened interrod zones (Figure 4C,D) are clearly seen. The central perforation upon acid etching is defined by the fact that the rod crystals are oriented with their long axis perpendicular to the front of the acid application so they will be etched more readily than the areas where crystals are oriented parallel or obliquely to the same front.

**Figure 5:**
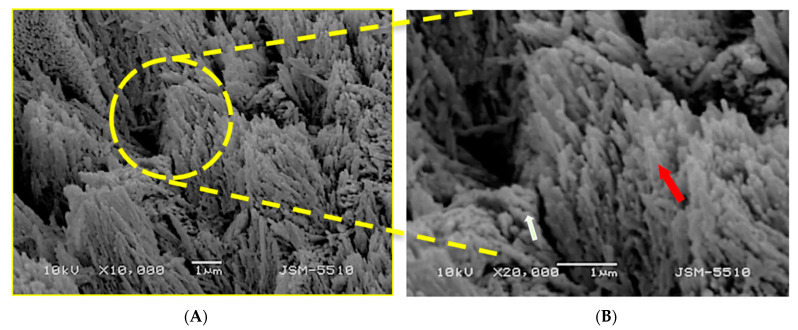
SEM pictures of demineralized enamel, made at two different magnifications (**A**,**B**), revealing: the rods’ dissolved walls (red arrow in Figure 5B) and high-roughness surfaces resulting from the irregular, anisotropic dissolution (white arrow in Figure 5B).

Upon the further acid spreading, the central part of the rods continues to dissolve and the interrod zones widen (Figure 5).

There is an enlargement of the interrod spaces due to the demineralization, forming pores between the rods and interrods and widening the intercrystalline spaces within rods (red arrow in Figure 5B). Nevertheless, some crystals in the rods remain in close contact, preferentially oriented alongside the prisms’ c-axis, keeping their original tightly packed arrangement (Figure 5).

##### SEM Characterization of Artificial Caries Lesions Remineralized with Hybrid PDMAEMA/Carbomer 940/CaP Microgels

The remineralization potential of the novel hybrid PDMAEMA/Carbomer 940/CaP microgels was tested on lactic acid-demineralized enamel (artificial caries lesions). According to the literature, when applying a remineralizion agent on caries lesions, initially, a continuous ACP layer is formed on the HAP crystals, which is further transformed into a crystalline HAP via a process known as epitaxial solid state transition [30,31,32]. Our strategy is aligned with this advanced biomimetic concept where the mineral clusters and amorphous particles act as an active base for the formation of the building blocks in the remineralization process [33]. Having identified the composition of the hybrid PDMAEMA/Carbomer 940/CaP microgels (via NMR and IR studies), we expected that the mixture of different CaP forms, such as amorphous TCP, HAP, ACP, OCP and/or DCPD, would ensure the effective remineralization of the artificial caries lesion. It is well known that HAP could be obtained from each of the phases identified by NMR in the PDMAEMA/Carbomer 940/CaP microgels, namely:

- directly from ACP (ACP→HAP) through direct structure reconstruction with partial dissolution that takes place during maturation;
- the transformation of OCP to the HAP phase can proceed either by a dissolution–precipitation reaction, or by a direct solid transformation. The structural similarity of the OCP and HAP structures enables HAP to grow epitaxially on the (100) surface of OCP [34];
- via dissolution and precipitation from DCPD (DCPD→HAP); as well as
- via β-TCP due to its excellent bioresorption.

Thus, one could expect very effective remineralization due to the availability of so many possible remineralization pathways.

**Figure 6:**
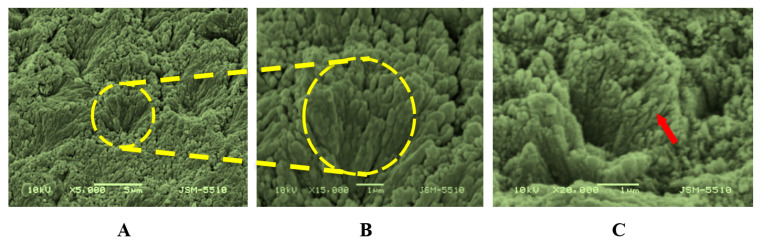
Newly formed CaP particles with various sizes and shapes on the top of the etched enamel rods: (**A**) and (**B**) continuous layer of deposited CaP on the surface of the demineralized enamel; (**C**) CaP nanocrystals grown on the demineralized enamel rod core (red arrow).

In Figure 6, the morphological features of the remineralized enamel samples with hybrid PDMAEMA/Carbomer 940/CaP microgels are presented as revealed by SEM. We should emphasize that prior to the SEM observations, the samples were washed thoroughly by water as well as by applying ultrasound treatment to ensure the removal of any hybrid microgel particles left attached on the remineralized enamel lesion.

Different zones of the remineralized enamel surface are seen: (i) rough and heterogeneous structure, the result from the demineralization (Figure 6A), as well as (ii) newly formed aggregates from spherical CaP particles with diameter ~20 to 50 nm on the demineralized surface (Figure 6C).

Moreover, the interface between the regenerated CaP particle layer and the underlying enamel shows a tight agglomeration and fusion, i.e., there is a seamless interface between the regenerated CaP layer and the underlying enamel (Figure 6B). Notably, the CaP particles are not haphazardly distributed, but their orientation is roughly perpendicular to the surface of underlying enamel (Figure 6C, red arrow). The microstructure of the newly formed layer is morphologically similar to that of the underlying natural enamel. We hypothesize that the active centers formed during the enamel dissolution (Figure 5 and Figure 6) could act as sites and preferential zones for epitaxial growth of the novel CaP phases which results in this seamless stitching between both CaP layers—the existing one and the newly grown one. We go further by suggesting that the polymer particles attach to the tooth surface due to their positive charge, while the tooth surface is known to be negatively charged. When attached to the enamel, they continue to release calcium and phosphate ions, thus enhancing and prolonging the process of the enamel remineralization, concomitantly guiding the preferential orientation of CaP formed on the demineralized enamel. In order to support our presumption, we have studied the release profiles of calcium and phosphate ions from hybrid PDMAEMA/Carbomer 940/CaP microgels in vitro (Figure S3). We have studied calcium and phosphate ion release over 6 h periods as this was the time length of the remineralization studies. The results confirm the ability of the newly developed hybrid materials to constantly release both ions, ensuring a fresh supply to the lesion surface where remineralization took place. Thus, the hybrid PDMAEMA/Carbomer 940/CaP microgels show good potential and could be successfully used as a new active enamel remineralization system.

If one compares the morphology of the remineralized enamel surface shown in Figure 6 and some similar remineralization studies, it could be concluded that the remineralization here mainly takes place on the top of the HAP rods, thus upgrading and restoring the etched enamel structure. In contrast, some remineralization studies performed with, e.g., agarose hydrogels on the demineralized enamel immersed in phosphate solution [35] resulted in filling up only the interrod spaces during the first 48 h of the remineralization. In contrast, the novel hybrid materials, developed within this study by taking advantage of the active centers of the demineralized HAP rods, were able to build them up along the c-axes via a constant supply of calcium and phosphate ions for the 42 h long remineralization process. This ensured seamless bonding between the HAP rods etched during the demineralization and the newly formed CaP via the remineralization process as clearly shown in Figure 6.

Similar “upgrading” of the HAP rods along their c-axes was observed in the case of demineralized enamel immersed in remineralizing solution of CaCl_2_, KH_2_PO_4_, NaCl and NaF, i.e., fluoride-based remineralization [36]. After treatment for 7 days in total, although crystallographically similar, the microstructure of remineralized enamel totally differs from the healthy enamel as the large plate-like crystallites deposited formed a layer over the enamel crystallites. Thus, although ensuring HAP crystallization over the demineralized HAP rods, this study did not result in morphology similar to the healthy enamel, in contrast to our results after less than 2 days (42 h) in which we observed HAP rods increase along their c-axes.

Our results are in line with the recently reported repair of root enamel via remineralization with epitaxial growth of calcium phosphate ion clusters (CPICs), stabilized with triethylamine [33]. The authors applied a drop of ethanol solution containing the stabilized CPICs, air-dried it and then immersed it in simulated oral fluid for 48 h. The introduction of CPICs produced an ACP precursor layer which gradually evolved to HAP on the enamel and, eventually, a well-crystallized HAP layer was observed. In this way, they achieved a precise reconstruction of the enamel structure from the nanoscale to the macroscale. The morphology attained by the present study is very close to the one that Schao et al. [33] have reported, probably due to the presence of ACP nanoclusters in the PDMAEMA/Carbomer 940/CaP hybrid microgels, however, the polymeric part in our case slowed down the remineralization process.

Thus, we could conclude that the idea explored in the current study for hybrid microgels as a delivery system for calcium and phosphate ions that ensures their prolonged release could result in visible remineralization of demineralized enamel.

#### 2.2.2. X-ray Diffraction of De- and Remineralized Enamel Lesions

**Figure 7:**
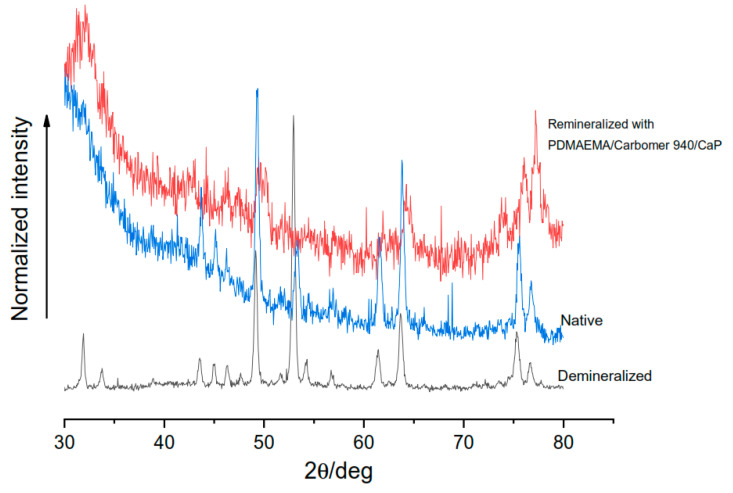
XRD patterns of native enamel, demineralized enamel and enamel remineralized with PDMAEMA/Carbomer 940/CaP.

The newly formed remineralized layer was studied by X-ray in an attempt to identify its composition and qualitatively estimate its crystallinity. The diffractograms of native enamel, demineralized enamel and enamel remineralized with PDMAEMA/Carbomer 940/CaP microgels are shown in Figure 7. The main peaks observed for the three samples are in good agreement with those in the XRD pattern of hydroxyapatite, JCPDF card #01-074-0565, thus indicating that the predominant component of the inorganic phase in the three samples is HAP. The sharp and intense 004 peak at 53.22 deg indicates that the (001) planes are parallel to the surface [37], i.e., the HAP crystals are aligned along the c-axis—an orientation which can also be seen in the SEM images for demineralized and remineralized samples (Figure 4, Figure 5 and Figure 6).

The crystallite size in the direction perpendicular to the (502) planes was numerically determined by using the Scherrer formula for the reflex well-expressed in all three samples at 2θ = 63.70 (Table 1). This peak was chosen as it was relatively strong and unaffected by adjacent peaks for all three samples. The results are presented in Table 1. While the crystallite sizes in the native and demineralized samples are close to each other, the crystallite size in the PDMAEMA/Carbomer 940/CaP-remineralized sample is significantly (x 2–3) smaller—a result from the conducted process of remineralization. This could be explained by the fact that the remineralized layer consists of newly grown CaP crystallites, formed on the artificial enamel lesion by using the etched HAP crystals as nucleation sites and the hybrid microgels as a source for Ca and P. The small crystallite size after the remineralization worsens its diffraction pattern (Figure 7) and results in low quality of the X-ray pattern for the remineralized sample as compared to the other two.

The observed crystallites could not be PDMAEMA/Carbomer 940/CaP hybrid microgels simply attached to the enamel as the hybrid microgels do not contain crystalline but amorphous CaP. Moreover, if such attachment happens it should result in much more chaotic assembly of CaP on the enamel surface—something which is not seen in the SEM images of the remineralized artificial caries lesion (Figure 6). Additionally, as was mentioned above, thorough washing and ultrasound cleaning of the remineralized teeth were performed in order to detach such hybrid microgels and be able to observe and study only the remineralized surfaces.

#### 2.2.3. Infrared Spectroscopy Characterization of Remineralized Artificial Caries Lesions

**Figure 8:**
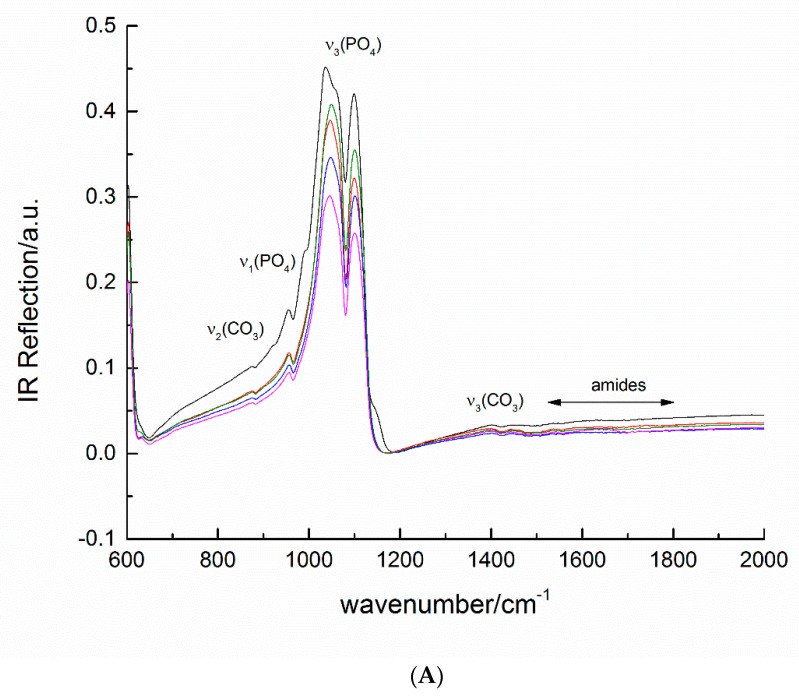

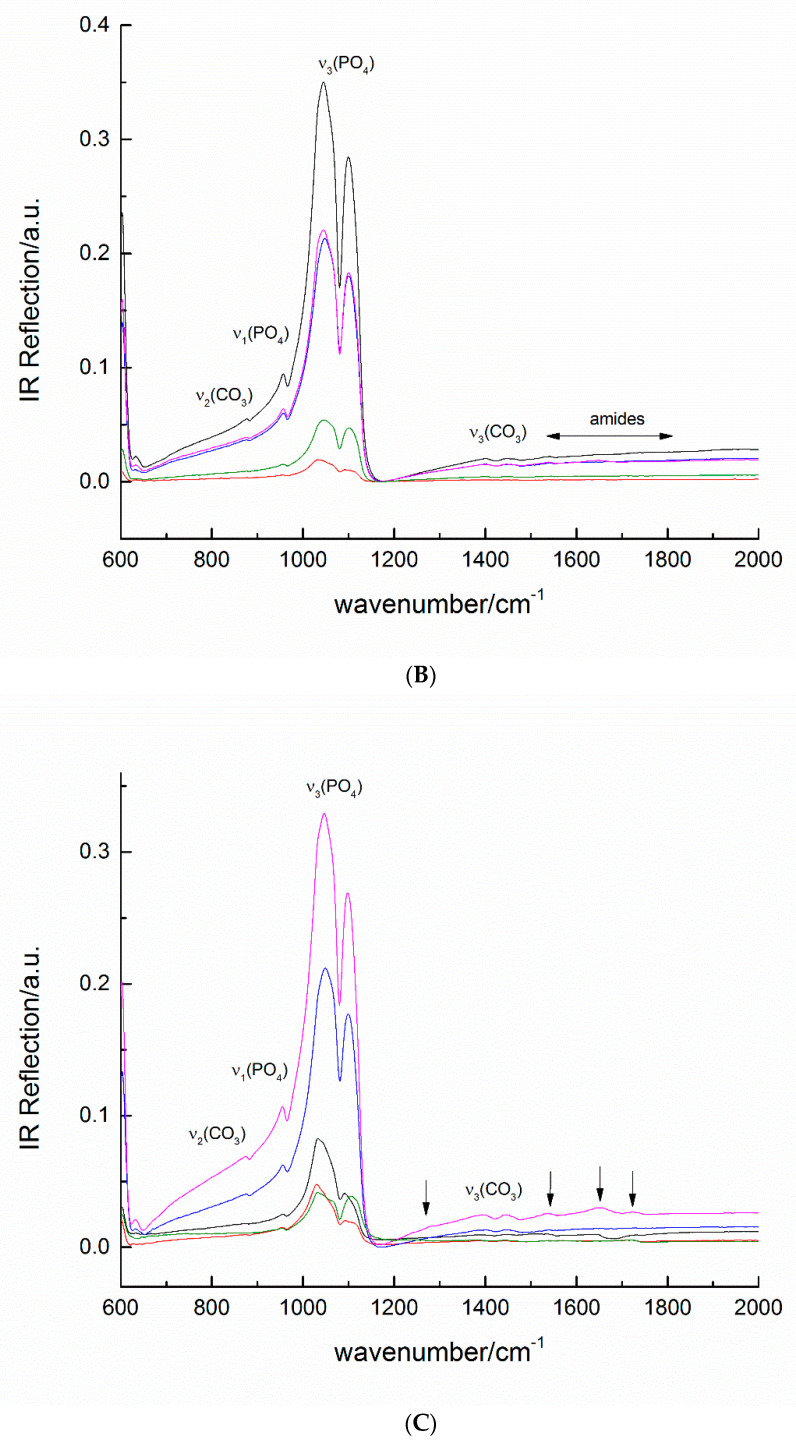
Infrared spectra of (**A**) native, (**B**) demineralized and (**C**) remineralized enamel (all figures present the spectra obtained for 5 independent samples) (the different spectra, designated with different color, are obtained for different samples, treated in the same way).

In Figure 8, the IR spectra of native, de- and remineralized enamel are presented. The infrared spectra of native enamel surfaces (Figure 8A, several spectra are presented as different samples of remineralized surfaces were studied for sake of comparison) reveal the most intensive peaks at 1040 and 1100 cm^−1^ arising from the antisymmetric stretching mode of phosphate groups, typical for enamel and carbonated hydroxyapatite [38,39].

In demineralized samples (Figure 8B), there is almost no change in the positions of the enamel peaks, however, a decrease in the total reflection intensity is observed which is due to the demineralization and the increased surface roughness as compared to the native enamel. The spectra confirm that the depth of demineralization does not reach the dentine. In some spectra, the most pronounced peaks are in the area of carbonate stretching vibrations in the range 1380–1550 cm^−1^ and of the amides at 1650 cm^−1^ which could be explained by the dissolution of the surface enamel layer. The detailed assignment of all infrared peaks is presented in Table S2.

The infrared spectra of teeth with remineralized enamel lesions are shown in Figure 8C. The most intense peaks of the phosphate group in hydroxyapatite shifts from 1045 cm^−1^ in the demineralized sample to 1026 cm^−1^ in the remineralized one as well as from 1100 cm^−1^ to 1120 cm^−1^. Such a shift is in the range of (PO_4_) stretching vibrations and could be explained by the change in the apatite surface layer and in situ precipitated phosphate phases [40,41]. Other, much less intense, peaks that do not belong to enamel are registered at 1226, 1650 and 1714 cm^−^^1^. They may be due to both the DCPD phase and the polymer (Table S2), however, as their intensity is very low compared to the HAP-related bands, these components are also very low in content.

#### 2.2.4. Raman Spectra of the Remineralized Artificial Caries Lesions

**Figure 9:**
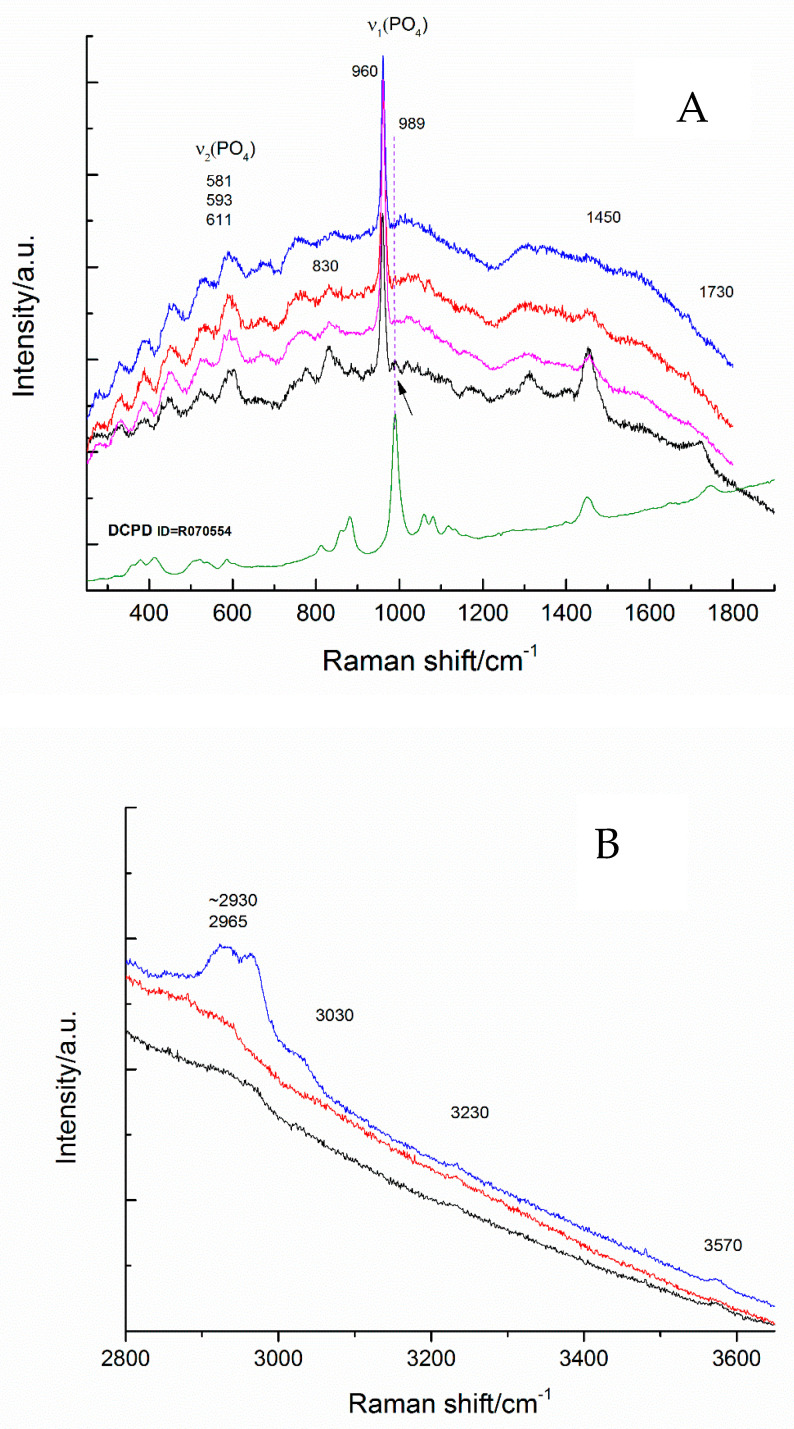
Raman spectra of remineralized enamel surfaces in different spectral ranges.

Raman spectra of remineralized caries lesions were also obtained for more detailed characterization of the remineralization process. The most intense signal was found to arise from the symmetric stretching of (PO_4_), which gives narrow peaks clearly distinguishable for different phosphate phases (Table S2). The position of the peak of symmetric stretching mode of the phosphate group in hydroxyapatite at 960 cm^−1^ remains unchanged (Figure 9A). The signal near 3000–2900 cm^−1^ (Figure 9B) appears in the range of C-H and C-H-N stretching and CH_2_ bending at 1459 cm^−1^ and could indicate the presence of a hybrid organic/inorganic layer on the enamel surface.

Newly formed calcium phosphates are difficult to detect due to the weak signal of these nanosized particles as compared to the hydroxylapatite from the artificial lesion. A very weak signal at 987 cm^−1^ coincides with the position of the most intense Raman peak of the phosphate group in DCPD (brushite). The other intense peaks of brushite overlap those of the organic component at 1740 and 2900–3000 cm^−1^ due to C=O and C-H vibrations, respectively (Table S2).

The newly developed PDMAEMA/Carbomer 940/CaP hybrid microgels have also demonstrated antibacterial activity which is very similar but weaker as compared to the neat PDMAEMA (see Supplementary Information, Table S3) (the different spectra, designated with different color, are obtained for different samples, treated in the same way).

## 3. Conclusions

The present study revealed the synthesis and characterization of novel PDMAEMA/Carbomer 940/calcium phosphate hybrid microgels and their application for remineralization of artificial caries lesions, aiming to repair the damaged enamel structure. The in situ calcium phosphate deposition in the novel core–shell PDMAEMA/Carbomer microgels was shown to result in the formation of mainly amorphous CaP, however, other polymorphic forms of CaP were also registered. This is one of the first studies that shows the influence of the polymer matrix on the calcium phosphate phases obtained via an in situ deposition process. In this respect, NMR appeared to be a powerful method for CaP phase evaluation as it allowed us to identify several CaP phases despite their highly amorphous nature, namely TCP, HAP, ACP, OCP and/or DCPD. All calcium–phosphate phases identified in the novel hybrid microgels, under appropriate conditions, e.g., pH, calcium and phosphate ion concentration, Ca/P ratio, etc., could serve as a source for calcium and phosphorus ions needed and used for the in situ formation of stable apatite, i.e., to ensure a constant supply of these ions for the successful enamel remineralization.

The remineralization of demineralized enamel resulted in the formation of new HAP crystallites on the top of the enamel rods. Moreover, the formed small HAP crystallites were aligned along the rods’ c-axis and thus present a new remineralized surface which reconstructs the artificial carious lesion. The morphological characteristics of the remineralized enamel samples treated with the novel hybrid materials, which are very close to the natural enamel, confirm the potential of the applied approach. Thus, the potential of the novel PDMAEMA/Carbomer 940/calcium phosphate hybrid microgels for remineralization and new CaP layer formation on the artificial caries lesions is demonstrated. The study also revealed that the approach to “deliver” a constant flow of calcium and phosphate ions via an appropriate Ca/P delivery system is a powerful approach to attain the remineralization and reconstitution of caries at the early stages of development.

## 4. Materials and Methods

### 4.1. Materials

Carbomer 940 (Carbopol^®^ 940, Lubrizol, Wickliffe, OH, USA, Scheme S1) was received as a donation from the Laboratory on Pharmaceutical Technology and Biopharmacy, Faculty of Chemistry and Pharmacy, Sofia University. 2-Dimethylamino ethylmethacrylate, anhydrous calcium dichloride, (CaCl_2_), potassium persulfate (K_2_S_2_O_8_), tetramethyl ethylenediamine (TEMED), acetic acid and methacrylic acid were purchased from Sigma-Aldrich, USA. Dipotassium hydrogen phosphate (K_2_HPO_4_), anhydrous, was purchased from Merck, Germany.

### 4.2. Preparation of the Remineralization System

#### 4.2.1. Synthesis of Linear PDMAEMA

Linear PDMAEMA (Scheme S1) was synthesized via free radical polymerization using 2M aqueous solution of 2-dimethylamino ethylmethacrylate monomer also containing 0.1 mol% initiator K_2_S_2_O_8_ and 0.2 *v*/*v*% TEMED (accelerator). The polymerization took place at room temperature (25 °C) for 24 h. At the end of polymerization, the reaction mixture was transferred into a dialysis tube (3.5K MWCO, 16 mm, SnakeSkin™ Dialysis Tubing, Thermo Scientific, Waltham, MA, USA) and immersed in distilled water to remove the non-reacted chemicals. The wastewaters were changed daily, and their purity was measured by a UV spectrophotometer, BOECO S-20, BOECO, Germany. The polymer solution, after its complete purification from residuals, was freeze-dried and the polymer, as a powder, was used in further experiments. The conversion of the monomer DMAEMA to PDMAEMA was gravimetrically determined to be 60%.

#### 4.2.2. Synthesis of PDMAEMA/Carbomer 940/Calcium Phosphate (CaP) Hybrid Microgels

**Scheme 1:**
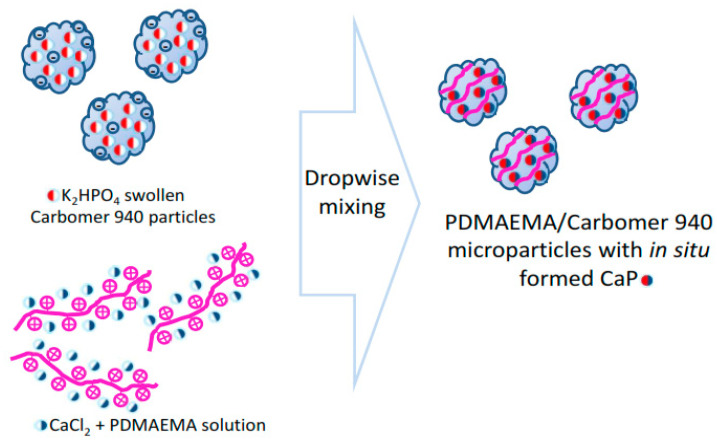
Preparation of hybrid PDMAEMA/Carbomer 940/CaP microgels.

The hybrid microgels were obtained via a one step procedure where a mixture of two solutions was prepared by slow (1 mL/min) dropwise addition of the *1st* solution to the *2nd* one using mild shaking (Scheme 1):

*1st solution*: 0.025 wt% PDMAEMA aqueous solution containing 0.0083 M CaCl_2_, prepared by mixing 50 mL 0.3 wt% solution of PDMAEMA in 1 M acetic acid with 10 mL of 0.5 M CaCl_2_ aqueous solution and diluted 10 times.

*2nd solution*: 0.025 wt% Carbomer 940 aqueous suspension containing 0.0047 M K_2_HPO_4_, prepared by suspending Carbomer 940 in distilled water to give a concentration of 0.3 wt.% and to 50 mL from this suspension, 10 mL of 0.28 M K_2_HPO_4_ aqueous solution were added. The obtained suspension was diluted 10 times and it remained opalescent, showing that the size of the obtained particles was above nanoscale.

The final weight ratio between the three main components of the hybrid microgels is PDMAEMA:Carbomer 940:CaP = 1:1:2 with Ca/P ratio = 1.77. pH of the suspension was measured immediately after its formation to be 5.6 (Hanna HI2210, Hanna instruments, Woonsocket, RI, USA).

### 4.3. Preparation of Demineralized Enamel Lesions

In the applied chemical model for creating an artificial dental caries, lactic acid is chosen as a demineralizing agent following the chemistry of the cariogenic activity of dental biofilms which is generally attributed to acid, and in particular to lactic acid, production by bacteria [42].

We used 30 non-carious erupted third molars, extracted for orthodontic purposes, to create artificial enamel lesions. All patients, aged between 20 and 30 years, provided written informed consent that they agree with the experimental study. The exclusion criteria were carious, fractured, restored, hypomineralized, fluorotic lesions and any other visible structural and mechanical enamel defects. After the artificial caries lesion formation, the treated tooth surfaces were manually cleaned of contaminants and surface debris using a periodontal curette, washed with distilled water and stored in 0.1% thymol solution at 4 °C until being used.

**Scheme 2:**
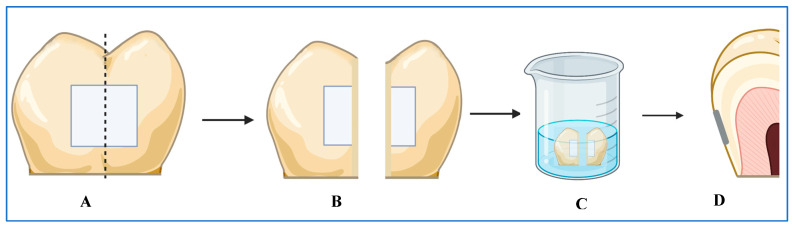
Tooth sample preparation technique: (**A**) the buccal half of the crown is covered with two layers of acid-resistant varnish, leaving a clean square window with dimensions 3 × 3 mm; (**B**) the buccal part is longitudinally cut into two halves and the cut surface is also varnished. Half **1** is used for evaluation of the sample’s demineralization and half **2** is used to evaluate the effect of the remineralization (frontal view); (**C**) demineralization of the samples for 6 days; (**D**) artificial carious lesion of enamel with depth < 100 µm (lateral view).

The preparation of the artificial caries lesions was carried out using the following procedure: the radicular part of each tooth was removed and then the crown was cut longitudinally by a diamond blade saw under water cooling to obtain buccal and lingual halves. A flat surface window of 3 × 3 mm in the middle of the equatorial surface section (Scheme 2A) was chosen for further treatment, while the buccal half was covered with two layers of acid-resistant varnish (Clearance, France). As demineralization is a function of many chemical and structural factors as well as of the direction and position of the acid attack [24], to closely mimic the subsurface enamel caries lesions, the aprismatic surface enamel layer was removed via fine polishing agent. This window was polished with 1 µm diamond suspension using SiC paper with grit sizes 320, 600 and 1200 (Shofu, Super-Snap Rainbow Technique Kit, Kyoto, Japan). Then, the buccal part was cut into two halves (Scheme 2B).

To carry out the demineralization, we used an aqueous solution of CaCl_2_ (2.2 mM), NaH_2_PO_4_ (2.2 mM), lactic acid (0.1 mM) and fluoride (0.2 ppm) with pH adjusted to 4.5. In this way, to create an artificial caries lesion we applied the chemical approach, which mimics the caries process through the use of acid or acid buffer but does not take into account the microbiological aspect of the caries formation [25]. The samples were immersed in this solution for 6 days, during which time the solution was renewed every 24 h (Scheme 2C).

After demineralization, the samples were washed in distilled water, sonicated for 5 min and stored at 4 °C in distilled water prior to use (Scheme 2D). The obtained two symmetrical halves originating from one tooth were used respectively to: (i) evaluate the demineralization process by scanning electron microscopy (SEM) and infrared spectroscopy and (ii) to realize the remineralization treatment. In this way, the demineralization and remineralization processes were always compared with two pieces from one and the same tooth.

### 4.4. Remineralization of the Artificial Enamel Lesions with PDMAEMA/Carbomer 940/CaP Hybrid Microgels

The demineralized tooth samples were carefully wiped to remove the excess water from their surface. Then, each sample was immersed in 6 mL of freshly prepared hybrid microgel suspension (the preparation procedure described in Section 2.2.1) and kept there for 6 h. It is known that the continuous remineralization in the oral cavity is restricted to the periods between dietary ingestion, i.e., between 1 and 4 h in the daytime or 6 and 8 h during the night. Thus, the remineralization time was fixed to 6 h in order to correspond to the oral habits (i.e., night sleep). After that, the sample was carefully rinsed with distillated water to remove the precipitates and other water-soluble components deposited on the tooth surface and then stored in 6 mL distilled water for the rest of the day, i.e., for 18 h, until the next application of freshly prepared fresh PDMAEMA/Carbomer 940/CaP suspension. This procedure was repeated 7 times, thus mimicking the remineralization of the artificial caries lesion for one week.

### 4.5. Scanning Electron Microscopy (SEM)

The morphology of demineralized and remineralized tooth surfaces was examined by scanning electron microscope (JSM-5510, JEOL, Tokyo, Japan) operating at 10 kV. Prior to the study, the samples were coated under an inert argon atmosphere for 30 s with gold using a sputter-coater (JSC 1200, JEOL, Japan).

### 4.6. Transmission Electron Microscopy

Freshly prepared suspension of PDMAEMA/Carbomer 940/CaP prepared as aforementioned was studied by transmission electron microscopy (TEM). To this purpose, the suspension was dropped on a copper grid. The grid was placed and examined by TEM (JEOL-2100, 200 kV) with energy dispersive X-ray analysis.

### 4.7. Infrared Spectroscopy (FT-IR)

Freshly prepared hybrid PDMAEMA/Carbomer 940/CaP microgels were immediately freeze-dried and the resulting powder was studied using a Tensor 37 (Bruker) FT-infrared spectrometer in transmittance mode after averaging 128 scans on standard KBr pallets at room temperature.

Dental specimens obtained either after demineralization or after the remineralization procedure were studied with a Hyperion 2000 infrared microscope in the spectral range 600–5000 cm^−1^ with 20× Schwarzschild objective in reflectance mode after accumulating 264 scans. Microinfrared spectra were collected from three different areas with mean size 100 µm^2^ for each sample.

Raman spectra of remineralized samples were collected using an HR LabRam (Horiba) spectrometer (600 grooves/mm grating) coupled with an Olympus optical microscope and 50× objective in the range 100–4000 cm^−1^. The 632.8 nm line of a He-Ne laser was used for sample excitation. The Origin 9 software package was used for spectral evaluation.

### 4.8. Nuclear Magnetic Resonance (NMR)

NMR spectra were recorded on a Bruker Avance III 500 NMR spectrometer operating at 500.13 MHz ^1^H frequency (202.46 MHz for ^31^P), using a 2.5 mm solid state CP/MAS dual ^1^H/X probe head. The samples were loaded in 2.5 mm zirconia rotors and spun at a magic angle spinning (MAS) rate of 15 kHz for all measurements. The quantitative ^31^P NMR spectra were recorded with *one* *pulse* sequence (Bruker Topspin library), 90° pulse length of 2.8 μs, 5K time domain data points, spectrum width of 59 kHz, 64 scans and a relaxation delay of 60 s. The spectra were processed with an exponential window function (line broadening factor 50) and zero filled to 8 K data points. The ^1^H→^31^P cross-polarization MAS (CP MAS) spectra were acquired with the following experimental parameters: ^1^H excitation pulse of 3.6 μs, 5 ms contact time, 5 s relaxation delay, 256 scans were accumulated, MAS rate was 15 kHz. A ^1^H SPINAL-64 decoupling scheme was used during acquisition of CP experiments. All ^31^P chemical shifts were referenced against the external solid reference NH_4_H_2_PO_4_ (δ 0.9 ppm). The DMfit software was used for the deconvolution, simulation and fitting of the experimental NMR data [43].

### 4.9. X-ray Diffraction (XRD)

Wide angle X-ray diffraction patterns were recorded using a Siemens (BRD) D500 diffractometer, operated in reflection, utilizing a secondary monochromator filtered CuK_α_-radiation. The crystallite size *D*_hkl_ (in Å) in the direction perpendicular to the (*hkl*) plane (502) was calculated according to Scherrer’s formula:

(1) $$D_{\langle 502\rangle }=\frac{0.9*\lambda }{\beta_{\frac{1}{2}}cos\theta },$$

where *λ* = 1.542 Å is the wavelength used, 2*θ* is the reflection position and β_1/2_ in rad is the half-width of the full height for the reflection *hkl* positioned at 2*θ*. Gaussian function was used to approximate the reflections.

## Figures and Tables

**Table 1:** Crystallite size of native enamel, demineralized enamel and enamel remineralized with PDMAEMA/Carbomer 940/CaP hybrid microgels.

Enamel Sample	Native	Demineralized	Remineralized
Crystallite size (Å)	153	184	72

## Data Availability

The raw/processed data required to reproduce these findings cannot be shared at this time as the data also form part of an ongoing study.

## Supplementary Materials

The following supporting information can be downloaded at: https://www.mdpi.com/article/10.3390/gels8100681/s1. Scheme S1. Structural formulas of the polymers used within the study. Figure S1. Calibration curve for the quantitative determination of the released phosphate ions using absorbance at 830 nm. Figure S2. Calibration curve for the quantitative determination of the released Ca^2+^ using absorbance at 830 nm. Figure S3. Release profiles of calcium and phosphate ions from the hybrid PDMAEMA/Carbomer 940/CaP microgels in water. Figure S4. Release profiles of calcium and phosphate ions from the hybrid PDMAEMA/Carbomer 940/CaP microgels in acetate buffer. Table S1. 31P chemical shifts of different calcium phosphate phases. Table S2. Raman and IR peak positions in different phosphate phases, polymer/CaP microgels and remineralized tooth surfaces (vs = very strong; w = weak). Table S3. Antibacterial activity of PDMAEMA and PDMAEMA/Carbomer 940/CaP microgels.
