# Supplementary material for: Bioinspired Remineralization of Artificial Caries Lesions Using PDMAEMA/Carbomer/Calcium Phosphates Hybrid Microgels
Source: Gels. 2022 Oct 21;8(10):681. doi: 10.3390/gels8100681 (PMC9601719; doi:10.3390/gels8100681)
Supplement: Supplementary file 1 [file gels-08-00681-s001.zip › gels-1939032-supplementary.pdf]

Supplementary Information

Structures of the polymers used within the study

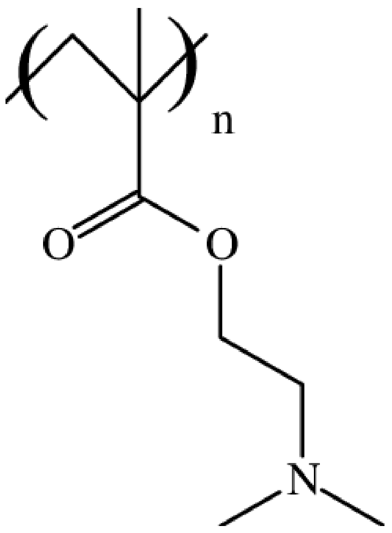

Poly[2-(dimethylamino)ethyl methacrylate
PDMAEMA

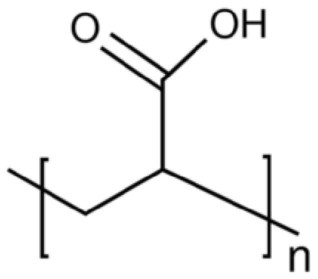

Poly(acrylic acid)
(Carbomer 940)

Scheme S1. Structural formulas of the polymers used within the study.

Calibration curves used for calcium and phosphate ions determination

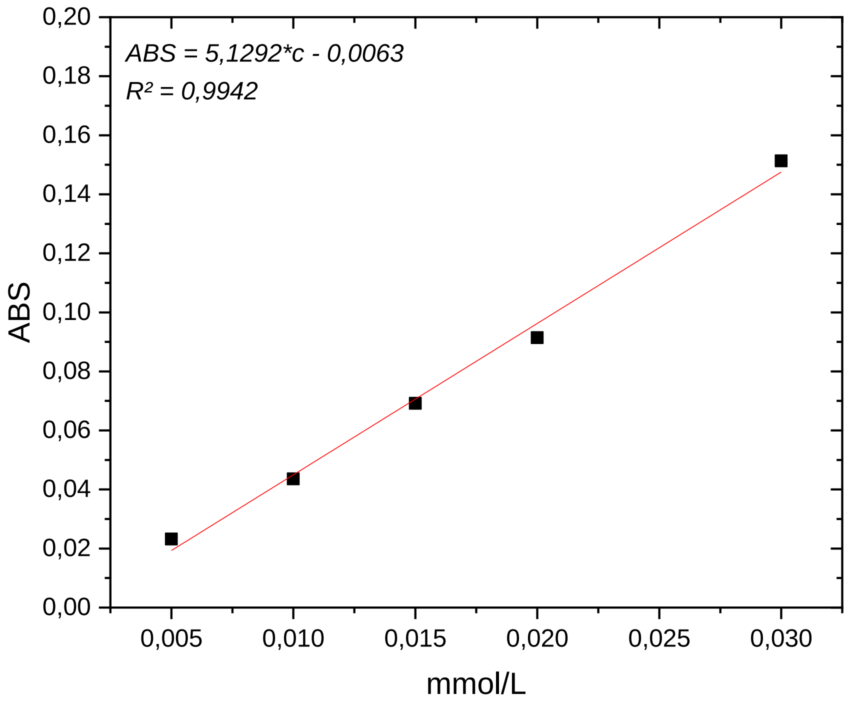

Figure S1. Calibration curve for the quantitative determination of the released phosphate ions using absorbance at 830 nm.

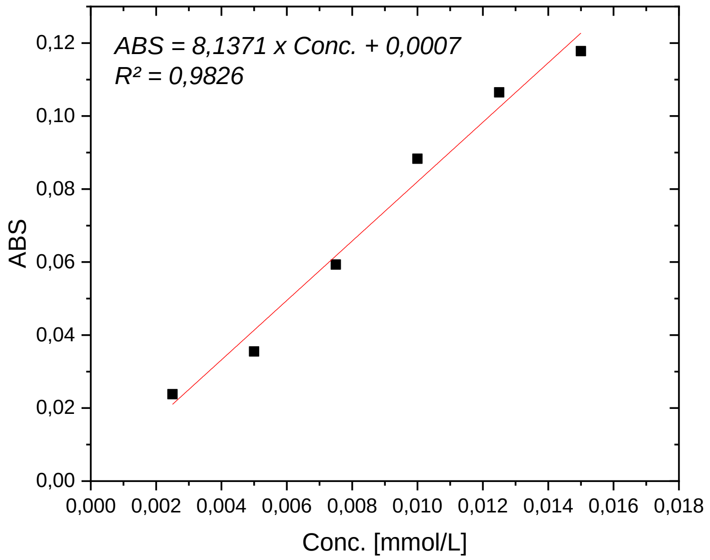

Figure S2. Calibration curve for the quantitative determination of the released Ca^{2+} using absorbance at 830 nm.

NMR characterization of different calcium phosphates phases

Table S1. ^{31}P chemical shifts of different calcium phosphate phases.

Phase	Isotropic ^{31}P chemical shift, ppm
monocalcium phosphate monohydrate<br>MCPM, $Ca(H_2PO_4)_2.2H_2O$	-4.6, -0.1<br>-4.2, 0.3
Dicalcium phosphate anhydrous DCPA, $CaHPO_4$ (monetite)	-1.5, 0<br>-1.5, -0.3/(-0.2)<br>-1.7, -0.1
dicalcium phosphate dihydrate (brushite)<br>DCPD, $CaHPO_4.2H_2O$	1.7<br>1.4<br>1.3
hydroxyapatite<br>HAP, $Ca_{10}(PO_4)_6(OH)_2$	2.8<br>2.9<br>2.3<br>3.3 (poorly crystalline HAP)
amorphous calcium phosphate<br>ACP, $Ca_xH_y(PO_4)_z \cdot nH_2O$	2.8<br>3.0
α-tricalcium phosphate α-TCP, $Ca_3(PO_4)_2$	0.5, 1.8, 2.2, 3.3<br>from -0.3 to 4.7, 12 peaks<br>from -0.4 to 4.0, 12 peaks
β-tricalcium phosphate<br>β-TCP, $Ca_3(PO_4)_2$	0.1, 1.2, 4.2<br>-0.5, 0.3, 4.3<br>0.1, 1.5, 2.8, 4.4, 5.5
octacalcium phosphate<br>$Ca_8H_2(PO_4)_6 \cdot 5H_2O$<br>$Ca_8(HPO_4)_2.(PO_4)_4.5H_2O$	-0.1, 3.4<br>-0.2, 2.2, 3.3<br>-0.4, 1.9, 3.1, 3.6<br>-0.2, 2.0, 3.3, 3.7<br>-0.5, 2.8

IR characterization of neat PDMAEMA/Carbomer 940/CaP microgels

The IR spectrum of PDMAEMA/Carbomer microgels reveals peaks characteristic for both polymeric components of the neat microgels, namely poly(acrylic acid) (Carbomer 940) and PDMAEMA (see Figure 3). These are as follows: the peaks at 2900-2700 cm^{-1} related to the stretching of C-H bonds; strong carbonyl peak at 1730 cm^{-1}; C-H bending at 1459 cm^{-1}, and peak at 1149 cm^{-1} which is in the range of C-O and/or C-N bonds.

Table S2. Raman and IR peak positions in different phosphate phases, polymer/ CaP microgels and remineralized tooth surfaces (vs=very strong; w=weak).

**DCPD**		**β-TCP**		**OCP**		**Enamel Carbonate-HAP**		**Polymer /CaP microgels**	**Remineralized tooth surfaces**		**Peak assignment**
**IR**	**Raman**	**IR**	**Raman**	**IR**	**Raman**	**IR**	**Raman**	**IR**	**IR Micro reflectio n**	**Raman**	
433		398<br>469	405<br>439<br>475<br>483	471<br>452	410<br>427<br>450	470	430<br>446	456		450	ν_2 (PO_4) bending
528				528				531			(ν_4) $(HPO_4)^{2-}$ bending
577		544<br>557<br>603	547<br>580<br>611<br>624<br>630	560<br>603<br>628	577<br>590	565<br>603	580<br>592<br>609	548<br>568<br>580		581<br>593<br>611	ν_4 (PO_4) bending
666<br>790						633		749		780	OH vibration
						873					ν_2 (CO_3) bending in enamel
876	878			874<br>914				858		830	P-OH stretching of $(HPO_4)^{2-}$ group
986	987(vs)<br>1000	940<br>976	948<br>961<br>970(vs)		957<br>966<br>(vs)	960(w)	960(vs)	988		960(vs)<br>987(w)	ν_1 (PO_4) stretching

					1010						
1060<br>1076<br>1137	1063<br>1085	1030<br>1060<br>1080<br>1100<br>1130	1016<br>1046<br>1084<br>1091	1023<br>1040<br>1058<br>1067<br>1075<br>1110<br>1126<br>1134		1035<br>1090<br>(vs)	1030<br>1049<br>1077	1044<br>1100<br>1116<br>1143	1026<br>1100	1025<br>1070	ν_3 (PO_4) stretching
								1143			C-O<br>C-N stretching
							1070<br>1100				ν_1 (CO_3) stretching
1218				1218<br>1297				1235<br>1270	1226		P-OH bending
						1420<br>1470<br>1550					ν_3 (CO_3) stretching
								1459<br>1470<br>1490		1459	C-H bending in CH_2
1650				1650							(ν_2) water bending
								1650<br>1730	1650<br>1714	1740	C=O stretching
								2714<br>2960<br>3400		2850<br>2930<br>2965<br>3030<br>3200	C-H and C-H-N stretching
2920<br>3164				3455							water stretching<br>HO-H

3290<br>3490<br>3544											
						3570	3570			3570	OH stretching in hydroxyapatite/ enamel

1226, 1650 and 1714 cm^{-1}

Release of calcium and phosphate ions from the hybrid PDMAEMA/Carbomer 940/CaP microgels

The dissolution tests for CaP from PDMAEMA/Carbomer 940/CaP microgels was performed in distilled water. To this purpose, 50 mg of the sample, containing ~25 mg CaP, was placed in 25 ml distilled water at 32°C. At defined time intervals (every 60 min), 3 ml aliquot of the dissolution media was withdrawn, filtered, and analyzed, then 3 ml fresh distilled water was returned.

The amount of Ca^{2+} ions was analyzed using an atomic absorption spectrophotometer Unicam SP 1950 in an air-acetylene flame. An aqueous solution of $CaCl_2{\cdot}2H2O$ with a concentration of Ca^{2+} ions 15 mmol was used as a standard. A calibration curve was also obtained by using Ca^{2+} ions concentrations in the range from 2 mg to 10 mg per L ($Y = -1.5 + 73.43x$; R2 = 0.99998).

The concentration of the phosphate ions was then determined by applying already known method. Briefly, in 10 ml volumetric flask to 1ml of the sample was added 1 ml aqueous solution of ammonium molybdate (11.38 mg/ml) and 0.4 ml aqueous solution of hydrazine sulphate (0.125 mg/ml) and then the volume was filled up to the mark with distilled water. The flask was kept for 30 min at 60°C then cooled down to room temperature (~25°C) and its absorbance was measured at 830 nm using UV spectrophotometer. Using the same procedure, a calibration curve was obtained for the quantitative calculations of the released phosphate ions. To this purpose, series of solutions with known concentration of K_2HPO_4 were prepared and their absorbance was measured in a similar way at the same wavelength. The calibration curve is presented in Figure S1. Results are presented on Figure S3.

In order to study the influence of pH and ionic strength on calcium and phosphate ions release profiles the dissolution of CaP was carried in a media containing sodium chloride (NaCl) solution (133 mmol/L) buffered to pH 5.5 with 50 mmol/L acetic acid proposed elsewhere. 50mg of the lyophilized powder was suspended in 100ml of media equilibrated at 32°C in beaker. The media was homogenized under magnetical stirring (100rpm). At different time points 1.5ml aliquots were withdrawn from the media and filtered through 0.45µm filter. Calcium ions concentration was determined by using UV/Vis spectrophotometry. First, the calibration curve was prepared using a stock solution of $CaCl_2$ (0.25mmol/L), sodium hydroxide (0.1mol/L) and murexide (0.6mmol/L). To this purpose, in 10ml volumetric flasks were mixed 1.2 ml of the murexide solution, 0.6 ml of the sodium hydroxide solution and

different volumes of calcium chloride solution. The volume was filled up to the mark with distilled water obtaining in this way colored solutions of Ca^{2+}-murexide chelate complex with different concentrations, respectively 0.015, 0.0125, 0.01, 0.0075, 0.005 and 0.0025 mmol/L with pH~11. A blank sample was prepared in the same manner without using Ca^{2+}. UV absorbance was determined at 498 nm wavelength. The calibration curve is presented in Figure S2. Again the phosphate ions concentration was determined as before. Results are presented on Figure S4.

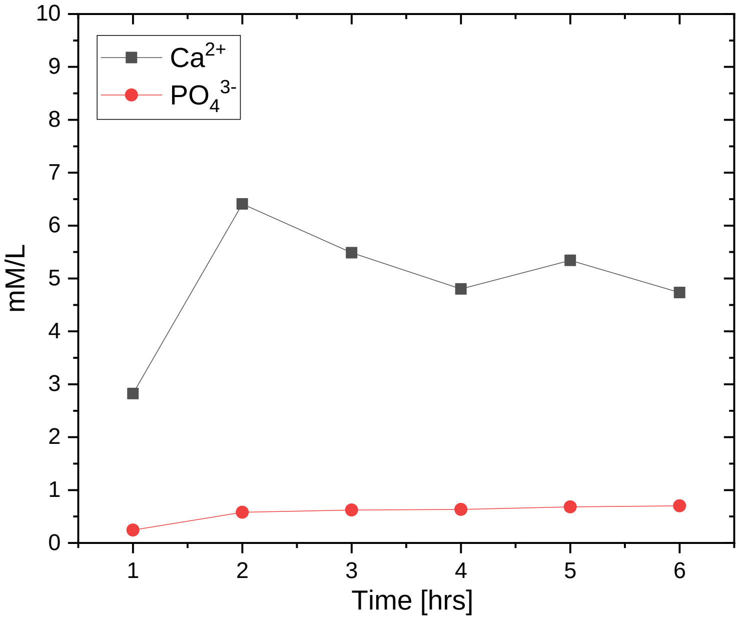

Figure S3. Release profiles of calcium and phosphate ions from the hybrid PDMAEMA/Carbomer 940/CaP microgels in water

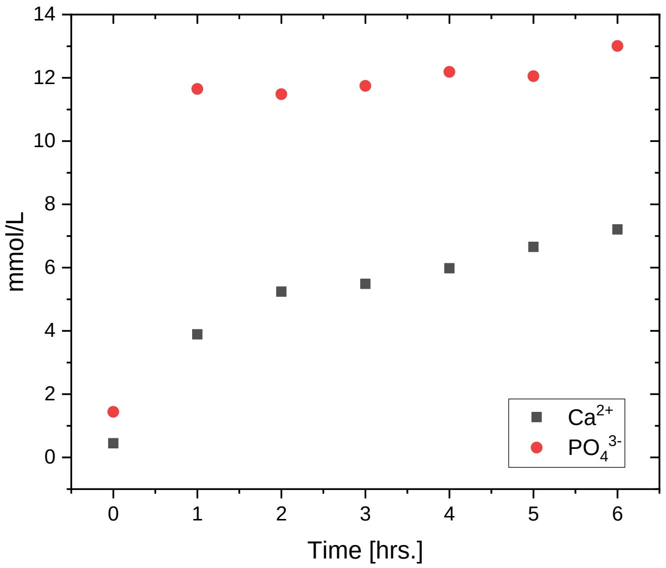

Figure S4. Release profiles of calcium and phosphate ions from the hybrid PDMAEMA/Carbomer 940/CaP microgels in acetate buffer

Antibacterial activity of PDMAMEMA and the hybrid PDMAEMA/Carbomer 940 /CaP microgels

Experimental part

The antimicrobial properties of the neat PDMAEMA and its hybrid PDMAEMA/Carbomer 940/CaP microgels were studied against the Gramm positive bacteria *Bacillus subtillis* and *Kocuria rhizophila*. A nutrient agar medium (NA, containing 0.1% w/v meat extract, 1% w/v peptone, 0.5% w/v NaCl, 1.5% w/v agar) was used. The PDMAEMA solution and PDMAEMA/Carbomer 940/CaP microgels suspension in water were used with concentration 0.05wt.%. The cultures were incubated at 28–30 °C for 24 h. A preculture of the *B. Subtillis* and *K. rhizophila* was grown in NA overnight at 28–30 °C. Each tested solution (20 µL) was added into the holes (d = 6 mm) made after solidification of the agar. The diameter of inhibition zones was measured on the 24th hour after the inoculation at 28– 30 °C. All equipment and culture media were sterile.

Results

The antibacterial activity of PDMAEMA and hybrid PDMAEMA/Carbomer 940 /CaP microgels suspension expressed as the diameter of the bacterial growth inhibition zone is presented in Table S3.

Table S3. Antibacterial activity of PDMAEMA and PDMAEMA/Carbomer 940 /CaP microgels.

**Bacterial culture**	**PDMAEMA**	**PDMAEMA/Carbomer 940/CaP**
B. Subtilis	10 mm	7 mm
K. Rhizophila	No activity	No activity

B. Subtilis and *K. Rhizophila* were chosen as being part of the oral microbiota, both of them being Gramm positive to which PDMAEMA is known to be less active as compared to Gramm negative bacteria. The amorphous calcium phosphates are not reported so far to exhibit antibacterial activity, except when in nanosized form, so for the hybrid PDMAEMA/Carbomer 940 /CaP microgels it is expected to possess bactericidal activity mainly due to the PDMAEMA in their composition. This was confirmed by the fact that the hybrid material exerted antibacterial activity against *B. Subtilis* slightly lower as compared to the neat PDMAEMA.

PDMAEMA has antibacterial activity against *B. Subtilis* because the latter has lipoteichoic acid anchored via a glycolipid into the outer leaflet of the cytoplasmic membrane. The acidic groups could provide sites at the bacterial cell surface to bind positively charged species, such as PDMAEMA,

allowing the latter to adsorb onto the cell membrane and to exert their bactericidal action. The way of action of the cationic biocides is suggested to abide to the following stages: (1) adsorption onto the bacterial cell surface through electrostatic interaction between the oppositely charged cationic biocides and the bacteria cell membrane, (2) diffusion of the biocides through the bacterial cell wall, (3) binding to the cytoplasmic membrane, (4) disruption of the cytoplasmic membrane, (5) release of cell cytoplasmic constituents, and (6) bacterial cell death. As PDMAEMA is a cationic polymer, the same mechanism of action is expected for it, namely adsorption onto the bacterial cell surface through electrostatic interactions and disrupting the cytoplasmic membrane through hydrophobic interactions. The hydrophobic interactions are known to be the reason for the antibacterial action of PDMAMEA against Gramm negative bacteria – the more pronounced the hydrophobic nature of the PDMAEMA based materials, the more successful are they against Gramm negative bacteria. However, the hydrophobicity does not appear to be as important in the inhibition of the Gram-positive bacteria as compared to the Gramm negative ones. The functional amine groups of PDMAEMA are often additionally quaternized in order to further enhance its bactericidal activity as they are capable of interacting with negatively charged bacterial cell surface structures.

Both, the neat PDMAMEA and the hybrid PDMAEMA/Carbomer 940 /CaP microgels do not exhibit any antibacterial effect against *K. Rhizophila* which could be explained by the lack of mycolic acids and teichoic acids in the bacterial cell walls which prevents the PDMAEMA adsorption and thus its antibacterial activity. This confirms our presumption that the antibacterial activity of the hybrid PDMAEMA/Carbomer 940 /CaP microgels is defined by PDMAEMA component.

The antibacterial activity of the newly developed remineralization system is very important for its planned application as it ensures stopping the negative effect of any bacteria presenting during the remineralization procedure which could prevent the effective caries treatment.
